# Assessment of Antifungal Pharmacodynamics

**DOI:** 10.3390/jof9020192

**Published:** 2023-02-01

**Authors:** Alex Howard, William Hope

**Affiliations:** Antimicrobial Pharmacodynamics and Therapeutics, Institute of Systems, Molecular and Integrative Biology, University of Liverpool, Liverpool L7 8TX, Merseyside, UK

**Keywords:** antifungal, pharmacokinetics, pharmacodynamics, translational, clinical, efficacy, animal

## Abstract

Pharmacokinetic-pharmacodynamic (PK-PD) analysis is of central importance to the progress of an antifungal agent into clinical use. It is crucial to ensure that preclinical studies give the best possible prediction of the way drugs are likely to behave in a clinical setting. This review details the last 30 years of progress in terms of disease model design, efficacy outcome selection and translational modelling in antifungal PK-PD studies. The principles of how PK-PD parameters inform current clinical practice are also discussed, including a review of how these apply to existing and novel agents.

## 1. Introduction

Establishing the efficacy of antifungal agents for patients with invasive fungal disease (IFD) underpins decision support for establishing in vitro susceptibility breakpoints and identifying regimens that confer optimal antifungal activity. The development of nonclinical models of IFD that are predictive of a clinical response to antifungal agents has been the focus of a concerted body of research. This review will consider the development of disease models, defined outcome measures and pharmacodynamic analyses used to accelerate and de-risk antifungal drug development.

## 2. Developing and Characterizing Disease Models

### 2.1. Host

The first critical step in establishing dose–exposure–response relationships involves the development of model systems that enable the interaction of a fungus with the host as it occurs in clinical settings. Since the early 1990s, the vast majority of in vivo antifungal pharmacokinetic-pharmacodynamic (PK-PD) studies have used either mice or rabbits, with comparatively fewer studies using rat, guinea pig, nematode or insect models [1,2,3,4].

Most laboratory animal models require some element of immunosuppression. This reflects the fact that most fungi are opportunistic human pathogens and most invasive fungal infections occur in immunocompromised patients. Furthermore, establishing invasive infection (e.g., mould infection via the respiratory tract) may be reliably achievable only in profoundly immunocompromised hosts. Immunosuppression has the additional advantage of providing a clearer picture of the activity of an antifungal drug against the pathogen without being confounded by innate and/or adaptive immunological responses.

The most common method of achieving immunosuppression in animal models has not significantly changed in the last 30 years. Cytotoxic agents (e.g., cyclophosphamide, cytosine arabinoside) and/or corticosteroids are administered via the subcutaneous (SC), intravenous (IV) or intraperitoneal (IP) route. In some but not all cases, broad-spectrum antibiotics are used in addition to prevent mortality from bacterial infections, but this is not invariably the case.

Towards the end of the 1990s, additional in vitro models of IFD were developed and characterised. Initially, these were limited to single-compartment mimics of bloodstream fungal infection that controlled the rates of inflow and outflow of drug-containing media at 37 °C to mimic the human concentration–time profiles of the antifungals being studied [5,6]. Subsequent in vitro models became more nuanced by mimicking anatomical sites relevant to fungal pathogenesis. For example, the construction of a cellular bilayer simulated the alveolar–capillary barrier that is transgressed in invasive pulmonary aspergillosis [7,8,9,10,11,12]. More recently, this approach has been modified by using nasal epithelial cells to mimic acute sino-pulmonary aspergillosis [13]. Such systems enable drug–pathogen interactions to be studied at high resolution and bypass welfare and resource considerations inherent to laboratory animal studies. Laboratory animal models, however, can better mimic factors relevant to clinical dose–exposure–response relationships, such as protein binding and anatomical compartments/barriers. The most theoretically robust approach is to use a combination of experimental models that yield complementary insights into antifungal pharmacodynamic relationships.

### 2.2. Pathogen Species

Most research has focused on *Candida* spp., *Cryptococcus neoformans* [14,15] and *Aspergillus fumigatus* [16,17,18]. More recently, other medically important fungal pathogens that represent an unmet medical need, such as *Candida glabrata, Candida krusei*, *Candida auris.* and *Aspergillus flavus*, have been studied to develop novel antifungal agents that address the challenge of innate and acquired antifungal resistance [13,19,20,21,22,23,24]. Each of these species varies in terms of its inherent pathogenicity in laboratory animal models. Intra-species pharmacodynamic variability is the norm, meaning that multiple strains of each species should be studied.

### 2.3. Minimum Inhibitory Concentration

The in vitro susceptibility of the challenge strain(s) to the test agent is critically important. This is typically determined using broth micro-dilution and the methodology of the Clinical and Laboratory Standards Institute (CLSI) and/or the European Committee on Antimicrobial Susceptibility Testing (EUCAST). For some drug–pathogen combinations, such as the echinocandins and fosmanogepix versus *Aspergillus* spp., a “clean” endpoint is not apparent. Hyphae appear drug-affected when they present morphologically stunted and aberrant forms. The minimum effective concentration (MEC) in this situation provides an alternative measure of potency. The MEC is defined as the lowest drug concentration where morphological changes occur that are indicative of abnormal growth.

The minimum inhibitory concentration (MIC) value for each drug–pathogen combination is a relative measure of in vitro potency. While the MIC is not an absolute value, as it is determined under specific test conditions, it usually provides useful biological information that serves in pharmacodynamic calculations—a two-fold higher MIC requires twice the drug exposure to achieve the same antifungal effect. Hence, the MIC provides a relative indication of antifungal activity and remains a mainstay of modern pharmacodynamic assessments.

Significant advances in the field of fungal genomics over the last 30 years have identified many of the genetic alterations underlying reduced susceptibility to antifungal agents. The most widely studied mutations in the preclinical PK-PD field involve the *fks* and *cyp51A* genes, which confer resistance to echinocandins and triazoles, respectively [23,25]. Antifungal efflux mechanisms and biofilm formation, however, may also be clinically relevant [26,27]. Despite the high correlation between these mechanisms and shifts in MIC and clinical efficacy, the MIC values obtained with wild-type populations still dominate pharmacodynamic analyses.

### 2.4. Pathogenesis

There has been a progressive increase in the number of experimental models that are mimics of specific clinical contexts and drug–pathogen combinations. Several examples are summarised in Table 1.

To provide the best chance of generating clinically relevant PK-PD relationships, it is important that experimental platforms enable the antifungal drug to interact with its fungal target in a way that is a faithful mimic of human disease. Fungal dimorphism presents a significant challenge in fulfilling this goal. This is especially the case for moulds such as *Aspergillus* spp., *Lomentospora prolificans*, Mucorales, *Scedosporium* spp. and *Fusarium* spp. [18].

For many moulds, the lung is the primary site of infection and clinical disease. The inhalation of hardy environmental forms (conidia or spores) and their germination into hyphae (the tissue invasive forms) is mimicked in laboratory animal models via the instillation of conidial (or spore) suspension into the nares or directly into the endobronchial tree. A tail vein model of invasive aspergillosis with hematogenous dissemination was commonly used but the approach has been largely discontinued.

For *Candida* spp., invasive candidiasis most commonly manifests following translocation across the gut wall and into the bloodstream with subsequent hematogenous dissemination. This process is mimicked experimentally via the IV inoculation of yeasts, with the primary disease site being the kidney, or the central nervous system as occurs in haematogenous *Candida* meningoencephalitis (HCME) [24,38,39]. Catheter-associated candiduria models have also been developed [35,36].

The most common murine model of cryptococcal meningitis involves tail vein injection with subsequent dissemination to the central nervous system. Mice are inherently susceptible to *Cryptococcus* and no immunosuppression is required. Rabbits are inherently less susceptible and mild immunosuppression with corticosteroids and direct intracisternal inoculation of yeasts is required to reliably establish meningitis and encephalitis.

## 3. Defining Outcome Measures

### 3.1. Quantifying the Antifungal Effect

Several different measures can be used to characterise dose–exposure–response relationships. Survival is commonly used, but two major limitations of this endpoint are the conflation of antifungal drug efficacy and antifungal drug toxicity, and the potential impact of immunosuppression. The approach is also practically and ethically difficult because of animal welfare issues, as suffering may be severe [40,41]. Quantitative mycological methods are now increasingly favoured.

Before the expanded application of molecular and antigenic techniques, colony forming unit (CFU) quantification from organ homogenates was the mainstay of mycological endpoints [19,42]. CFU-based techniques are appropriate for yeasts (as they are for bacteria). CFUs have also been used as part of in vitro techniques to model the fungistatic and fungicidal activity of antifungal agents, given that there is some evidence of early therapeutic success in invasive candidiasis with fungicidal therapy [43,44]. Using CFUs to quantify the fungal burden of moulds is more problematic because the number of colonies does not necessarily correlate with tissue burden [40]. Some fungal species can also be difficult to culture in the laboratory, and prolonged incubation periods needed to elicit growth increase the probability of laboratory contaminants. Towards the end of the 1990s, antifungal PK-PD studies increasingly incorporated a histopathological examination of lung tissue in studies of pulmonary aspergillosis [41]. These methods demonstrated antifungal drug effects on fungi that were not elicited during in vitro culture, including hyphal shortening, swelling and vacuolisation. The subsequent use of fluorescence microscopy enabled the detection of other effects on the intracellular architecture, including the disintegration of nuclei and mitochondria [45].

The fungal-specific biomarkers galactomannan and 1, 3, beta-D-glucan (BDG) have been increasingly used to quantify the fungal burden. Galactomannan correlates well with hyphal tissue invasion in vivo and in vitro, and can be measured in serum, bodily fluid or tissue [1,2,11,39,46,47,48]. Galactomannan cross-reactivity can occur with other fungal species [49]. Galactomannan and BDG can be detected in a variety of matrices, including CSF [39] and may be used to delineate pharmacodynamic relationships.

Quantitative PCR (qPCR) can also be used to quantify the fungal burden of moulds in tissues (*Aspergillus* spp. and *Rhizopus* spp.). The assay is limited to tissue homogenates [1,2,50]. qPCR potentially detects different morphotypes, including ungerminated spores/conidia, which may contribute to fungal burden when quantified using traditional culture, but are not specifically relevant to invasive disease. Quantitative reverse transcriptase PCR, however, has value in detecting live fungal cells [51].

A combination of endpoints is an especially powerful approach to delineating antifungal dose–exposure–response relationships. A variety of quantitative, semi-quantitative and qualitative measures can be used, including the measurement of lung weight, pulmonary infarction scores, survival, histopathology and radiological assessments. Recent work has also combined positron emission tomography (PET) imaging with the detection of the fluorescent antibody-labelled *Aspergillus* species, providing detailed views of fungal invasion that could potentially better characterise treatment response patterns [52].

### 3.2. Setting Endpoints

Anti-infective PK-PD research has used a wide variety of endpoints to define “therapeutic success”. There is no consensus on what might be appropriate, and the approach used varies according to the drug–pathogen combination and context. An overly ambitious target may hinder development because the exposures required may exceed those for which safety is established, whereas an insufficiently ambitious endpoint may result in unnecessarily poor efficacy in human studies. One solution is the use of positive controls using licensed antifungals with information spanning preclinical-clinical PK-PD, clinical PK-PD and clinical outcomes [53].

### 3.3. Establishing the Dose–Exposure–Response Relationship

A central tenet of modern pharmacodynamics is that the shape of the concentration–time profile is a critical determinant of the antifungal effect. Antifungal drugs exhibit either concentration-dependent or time-dependent activity that can be quantified using one or more of three pharmacodynamic indices:

fC_max_/MIC: a concentration-dependent measure; the free peak concentration of drug achieved in a compartment during a dosing interval, divided by the MIC of the organism.

%fT > MIC: a time-dependent measure; the percentage of the dosing interval free drug concentrations that exceed the MIC. C_min_ (minimum free drug concentration during a dosing interval)/MIC is an alternative time-dependent index if %fT > MIC cannot be used.

fAUC/MIC: a combination of both concentration and time; the free area under the concentration–time curve (a measure of total drug exposure during the dosing interval relative to the MIC).

Deciding which of the above indices are relevant requires dose fractionation studies where the total daily dose is administered in various schedules (e.g., two half dosages q12h, etc.) [54]. This distinction is not always clear-cut; the activity of amphotericin B and the echinocandins has been shown to potentially fall under more than one of the above categories depending on the pathogen and model [55,56]. This potentially has implications for dosing duration (e.g., continuous infusion of amphotericin B) and frequency (e.g., increased dosing intervals in echinocandins) [57,58].

A deeper understanding of the pharmacodynamics of any drug–pathogen combination also requires the following considerations:

Plasma protein binding (PPB): This is usually measured by equilibrium dialysis and expressed as a percentage of the total drug concentration in plasma. The importance of PPB varies depending on the drug and the context. Generally, only the free fraction of the drug is considered pharmacologically active. The concentration and activity of plasma proteins are dynamic variables affected by many different biological factors, presenting a potential challenge to the relevance of in vivo and in vitro models in studies with patients. There is wide variability within the antifungal PK-PD literature as to whether total drug concentration, free drug concentration or both are measured and considered in analyses. The availability of a free drug is also complicated by complex binding patterns (e.g., amphotericin B deoxycholate [59]) and the fact that most antifungal agents are extensively and tightly bound, meaning that small errors in measurement can profoundly affect the assessment of the free fraction. These issues are largely unresolved.

Tissue partitioning: drug concentrations at the effect site are usually estimated using tissue homogenates, with matrix-assisted laser desorption/ionisation mass spectrometry imaging (MALDI-MSI) increasingly applied.

Active metabolites: Some antifungal compounds owe their activity entirely to active moities split from prodrugs (e.g., isavuconazonium sulphate [60]). Others, however, are converted to active metabolites with comparable potency to the parent (e.g., itraconazole). These may occur at concentrations that are pharmacologically relevant and need to be considered when establishing dose–exposure–response relationships [61].

Antifungal resistance: The development of fluconazole heteroresistance has been found in C. neoformans when exposed to the drug in vitro. This effect is due to the subpopulations with aneuploidy that appear to be reversible once the drug is removed [62].

**Figure 1:**
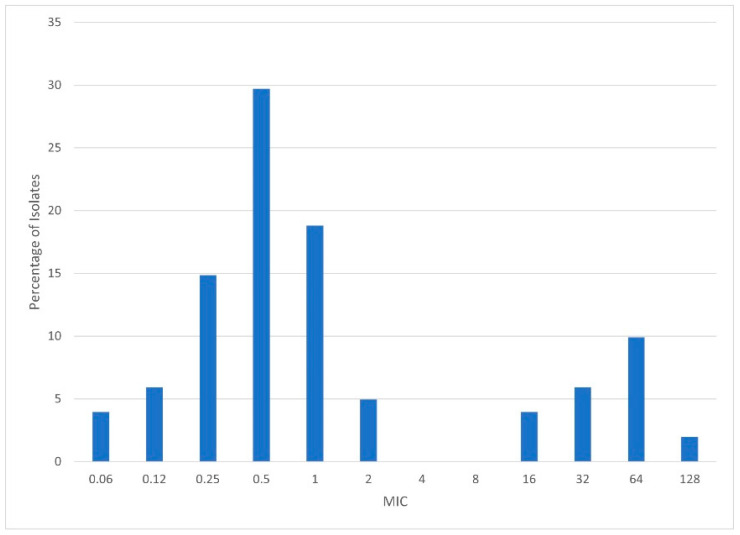
An example of an MIC distribution for an organism–drug combination, where the blue bars represent the percentage of isolates with each MIC in the population. The large left-hand cluster of MICs represents the wild type, and the smaller right-hand cluster represents organisms with an acquired resistance mechanism.

Separate considerations include characteristics affecting the practicality of use (e.g., oral bioavailability and the interactions with agents affecting drug-metabolising liver enzymes) [63,64]. Another key consideration is the MIC distribution among populations of each species of target pathogen. Preventing clinical failures necessitates coverage of the MICs found in wild-type populations. For the example shown in Figure 1, the regimen of choice will need to be active against organisms with an MIC of at least 2.

## 4. Regimen Identification

### 4.1. Human Pharmacokinetic Data

The identification of candidate therapeutic regimens for patients requires pharmacokinetic data from healthy volunteers, which should consist of single ascending dose (SAD) studies, followed by multiple ascending dose (MAD) studies. The design of these studies requires the attainment of the drug exposures predicted to have efficacy from preclinical studies, while simultaneously maintaining the safety margins identified in Good Laboratory Practice (GLP) toxicology studies [65,66].

### 4.2. Pharmacokinetic Modelling

A range of approaches are used to describe the pharmacokinetics of a new compound. Both noncompartmental and population methodologies may be used. The latter enables robust estimates of pharmacokinetic variability, which is critical for understanding the adequacy of a fixed regimen for a large population of patients. The variability in therapeutic response among IFD patients is typically larger than for healthy volunteers and this can lead to a smaller proportion of the patient population achieving drug exposures that are both safe and effective. Artificially inflating the variance for clearance may be necessary to obtain tractable estimates of the likely adequacy of candidate regimens. Increasingly, data from phase II and III studies contain embedded PK and PD studies that can be used to gain an insight into the adequacy of antifungal regimens as the clinical programs progress.

## 5. Conclusions

The outcomes of IFD remain poor, and many agents cause significant toxicity using the regimens required for therapeutic efficacy against populations of wild-type organisms. Fortunately, an increasing number of agents are being developed to address unmet medical needs, including triazole-resistant Aspergillus species and multi-drug-resistant *C auris*. PK-PD studies substantially de-risk the development of new antifungal agents by enabling initial estimates of appropriate dosing regimens for patients with IFDs.

## Figures and Tables

**Table 1:** Examples of preclinical systems used to model invasive fungal disease in vivo and in vitro.

Disease	Model(s)
Disseminated candidiasis	Murine tail vein injection, intravenous injection in rabbits
Haematogenous *Candida* meningoencephalitis	Intravenous injection in non-neutropenic rabbits
*Candida* intra-abdominal abscess	Murine intraperitoneal injection
Urinary catheter-associated candiduria	Urinary catheter lumen injection
Disseminated aspergillosis	Murine tail vein injection
Pulmonary aspergillosis	Murine nasal inoculation/aerosol chamber, rabbit endotracheal inoculation, in vitro models
Cerebral aspergillosis	Murine intracerebral injection
Sinopulmonary aspergillosis	In vitro and murine nasal inoculation
Cryptococcal meningitis	Murine tail vein injection and intracisternal inoculation in rabbits

References [10,13,14,24,28,29,30,31,32,33,34,35,36,37].

## Data Availability

Not applicable.
